# Employee informal coaching and job performance in higher education: The role of perceived organizational support and transformational leadership

**DOI:** 10.1371/journal.pone.0320577

**Published:** 2025-04-01

**Authors:** Giang Thuy Nguyen, Jana Matošková, Nhat Tan Pham, Man The Nguyen

**Affiliations:** 1 Tomas Bata University in Zlín, Zlín, Czech Republic; 2 Faculty of Economics and Business, Hoa Sen University, Hochiminh City, Vietnam; 3 School of Business, International University, Ho Chi Minh City, Vietnam; 4 Vietnam National University, Ho Chi Minh City, Vietnam; Southeast University, CHINA

## Abstract

The existing literature has not adequately explored the connection between informal coaching and employees’ job performance, particularly in higher education institutions (HEIs). Anchored in the social exchange theory, this study aims to investigate the impact of informal coaching practices on job performance, as well as the mediating and moderating roles of perceived organizational support and transformational leadership. Results from a time-lagged study of 768 participants in HEIs in Vietnam indicate that informal coaching from supervisors and peers is crucial in directly enhancing employee performance. Furthermore, the research indicates that perceived support from the organization plays a vital role in mediating the impact of informal coaching on individual job performance. Interestingly, the study suggests that high levels of transformational leadership strengthen the relationships between informal coaching from supervisors and employees’ job performance, as well as informal coaching from supervisors and perceived organizational support. However, contrary to expectations, transformational leadership does not moderate the influence of informal coaching from peers on job performance and perceived organizational support.

## Introduction

Coaching has received significant attention as a tool for supporting staff to realize their full potential in the workplace [1,2]. It is defined as a process that inspires a person to reach their potential [3]. Coaching can be formal or informal [4]. In formal coaching, the organization assign one official coach, usually the supervisor, to the coachee, with planned coaching procedures that include a timeframe, coaching activities, supervision, and a commitment to achieving specific goals [5] In contrast, informal coaching occurs during everyday workplace conversations. It is unplanned and spontaneous, such as a short highly focused coaching conversation in the corridor in the midst of a busy project [6]. Formal coaching involves structured and prearranged sessions while informal coaching is day-to-day activity that is unexpected [7]. Through coaching, employees can take time to set their own priorities and improve their life. In turn, it helps maintain a better work-life balance, improve employee relations, collaboration, and performance [8]. Despite this importance, existing literature on coaching, particularly informal coaching, still presents several research gaps.

Firstly, although workplace coaching in general has received attention from prior scholars [9,10], there is still a paucity of research on the specific roles of formal or informal coaching as tools for improving individual performance. The existing literature indicates that informal coaching is considered supportive of formal coaching, yet the impact of this approach on individual performance is still not well understood [6,11]. Thus, an empirical study on informal coaching aimed at clarifying its role in improving employee performance is needed [4], especially in the higher education institutions (HEIs). Specifically, although HEIs need to have talented faculty staff with high qualifications and effective job performance to deliver knowledge to students, they have not made informal coaching a high priority in their schemes [12]. Informal coaching has been suggested as a potentially effective substitute for formal mentoring relationships in faith-based higher education institutions [13]. This type of coaching leverages existing relationships and shared experiences among faculty members, fostering a collegial atmosphere where educators can freely exchange ideas, discuss challenges, and share pedagogical strategies. This method can be particularly beneficial for promoting continuous professional development and enhancing job performance, as it allows for immediate feedback and personalized advice. Moreover, informal coaching encourages the development of a supportive academic community, where faculty members feel valued and supported by their peers. This can lead to increased job satisfaction and a deeper commitment to the institution’s mission, ultimately improving both individual and organizational performance. Previous studies also indicate that coaching, in general, is an endeavor to guide and motivate teachers in teaching and learning to perform their obligations effectively, and that doing so improves teachers’ ability to teach and learn [14,15]. However, research on how informal coaching enhances faculty staff performance in HEIs remains an underexplored area. Therefore, this study is essential to fill this gap by investigating the link between informal coaching and employee performance in the higher education sector.

Secondly, prior studies have shown that general coaching is perceived as a form of organizational support, which may influence employee commitment and job performance [16,17]. Organizational fairness, support from leaders, human resource practices, support from colleagues, and work conditions are all forms of organizational support [18]. Anchoring in the social exchange perspective, employees view coaching activities, including informal coaching from managers and peers, as forms of organizational support [19], which in turn leads to higher job performance. Thus, it is expected that perceived organizational support (POS) could be an essential factor in the relationship between informal coaching and employees’ job performance. POS has been found to mediate the association between various workplace interactions, such as restructuring processes and job satisfaction [20], loyalty and organizational benefits, procedural fairness, and supervisor support [21], as well as organizational commitment and job satisfaction [22]. However, previous works have not fully investigated the mediation mechanism between informal coaching and job performance [23,24], particularly the mediating role of POS.

Finally, in the context of informal coaching, understanding the role of leadership (e.g., transformational leadership) is crucial, as leaders play a significant role in creating an informal work environment, that fosters mutual support among employees, generates optimal performance and productive outcomes [25]. Prior research stated that transformational leadership, defined as a type of leadership where “the leader moving the follower beyond immediate self-interests through idealized influence (charisma), inspiration, intellectual stimulation, or individualized consideration” (p.11) [26], inspires followers to exceed their performance expectation [27,28]. Also, it plays a crucial role in helping a leader as a representative of the company, convey the perception of organizational support to employees, which in turn fosters a high level of affective commitment to the company [29]. Previous research has identified the positive impact of transformational leadership on both coaching and employee outcomes, as well as its role as a moderator in the connection between human resource management practices and staff behavior [30], between teachers’ work engagement and receptivity to change in the field of education [31], and between age-based fault lines and perceived productive energy in a multinational corporation that produces construction tools and applications [32]. Despite the recognized importance of both informal coaching and transformational leadership in enhancing employee performance, the specific ways in which transformational leadership influences the effectiveness of informal coaching on job performance remain underexplored. Obviously, transformational leadership benefits for organizations in conjunction with informal coaching because a coaching program aligned with this leadership could effectively enhance leadership development and connect with employees’ performance [33] and employee development for the future [34]. Thus, exploring this research gap is essential.

In conclusion, this study addresses the following two questions: “How does informal coaching influence employees’ job performance in HEIs?” and “What are the roles of transformational leadership and POS in this informal coaching-job performance relationship?” By tackling these questions, this research makes significant theoretical and practical contributions. Theoretically, it advances the understanding of informal coaching by employing SET to examine its distinct impact on job performance within the underexplored context of HEIs. This framework not only elucidates the direct effects of informal coaching but also highlights the mediating role of POS and the moderating influence of transformational leadership, thereby addressing critical gaps in the existing literature. Furthermore, the study provides nuanced insights into the unique dynamics of informal coaching relationships, offering a deeper understanding of how such practices operate within organizational contexts. These findings extend the application of SET and contribute to the fields of coaching, leadership, and organizational behavior by clarifying the interplay between informal coaching, leadership styles, and organizational support.

Practically, the study underscores the value of informal coaching as a strategic approach to enhancing faculty performance and achieving institutional outcomes. This is particularly significant for HEIs in developing economies, such as Vietnam, where improving academic staff performance is essential for advancing educational and economic goals. Additionally, these insights hold broader implications for HEIs worldwide, illustrating the universal importance of fostering informal coaching practices and supportive leadership to drive organizational success. By bridging theoretical exploration with practical relevance, this study provides a comprehensive framework for understanding and implementing informal coaching in diverse higher education settings

## Theoretical background and hypothesis development

### Social exchange theory (SET)

This theory is a core theoretical framework for understanding human relations, founded on the concept of reciprocity in relationships [35]. According to Blau [35], social exchange is “the voluntary actions of individuals that are motivated by the returns they are expected to bring and typically do in fact bring from others” (pp. 91-92). A key concept of social exchange theory is that individuals offer favors or benefits to others with the expectation of receiving something in return. It refers to the exchange of activities, which can be tangible or intangible, involving rewards or costs between at least two individuals [36]. The Social exchange theory is frequently utilized to understand the outcomes of coaching interactions [37,38]. When an individual in an organization acts as a formal or informal coach, the coachee’s perception of these actions can vary. While such actions may be intended as goodwill, they may not always be perceived this way by the coachee, especially in the case of unsolicited informal coaching. It’s important to consider that the coachee’s perception of the coacher’s motivation plays a crucial role in determining the effectiveness of the coaching interaction. If the coaching is perceived as intrusive or unnecessary, it could lead to resistance or a lack of engagement. Therefore, understanding the context and the coachee’s perspective is essential in ensuring that informal coaching is well-received and effective [39,40]. Encouraging a culture of mutual support among employees, where questions are answered and supportive guidance is provided among colleagues, may increase the frequency of peer coaching activities. The perceived benefits of formal or informal coaching interactions motivate personnel to reciprocate by putting more effort into their work and improving their performance within the organization [41,42].

This study assumes that a more comprehensive understanding of the impact of coaching activities on job performance can be obtained by grasping the mediating function of the POS. According to the findings of Eisenberger [43], employees’ beliefs about their organization’s commitment to them, encouraging a culture of mutual support among employees (known as POS), play a key role in their own commitment to the organization. This means that when employees perceive high levels of POS, they feel a strong sense of obligation to be committed to their employers, which in turn motivates them to engage in behaviors that benefit the organization such as improved performance. By applying social exchange theory, this study addresses a significant gap by hypothesizing that effective informal coaching, supported by the organization, can influence employees’ perceptions and motivate them to reciprocate by putting in more effort to enhance their performance. In the context of this study, employee job performance and POS represent two reciprocal behaviors. Furthermore, Stinglhamber [29] stated that the leader, as a representative of the organization, conveys to followers through transformational leadership that the organization has treated them favorably, thereby raising perceived organizational support and, ultimately, affective commitment. This study also hypothesizes that transformational leaders can enhance the social exchange between the coach and coachee by providing additional resources, encouraging mutual support among employees and offering support beyond the coaching-based relationship [44,45].

### Coaching in the workplace

There is often confusion about the differences between formal coaching, informal coaching and mentoring so it is crucial to recognize the distinctions between them [46]. Coaching, in general, is defined as a goal-oriented approach where a coach works with an individual to develop abilities, boost performance, or meet predetermined goals [47,48]. It frequently focuses on the present and future, providing practical advice and constructive feedback to enable the coachee to realize their full potential [49,50]. Unlike formal coaching, which is structured and agenda-driven, informal coaching occurs spontaneously within everyday interactions, offering real-time support tailored to the coachee’s immediate needs [4]. Informal coaching can occur among colleagues, or between colleagues and their supervisors, and may take place in conversational dialogues in hallways, the break rooms, or through phone or video calls [51]. When people confront challenges in their career path, informal coaching is often needed and beneficial [8,52], as it allows staff to consult with various information sources to help them resolve their problems at any time [4].

On the other hand, mentoring involves a long-term relationship in which an experienced person provides guidance, support, and wisdom to a less experienced individual [53]. In mentoring, “the mentor is assumed to be highly experienced in the discipline or field in which the mentee is working, and in the workplace” (p.250) [54]. The mentors advocate for their protégés’ well-being by offering advice, recommendations, support and feedback as well as sharing knowledge based on their experience and what has worked well for them [55]. In contrast, in formal or informal coaching, the coachee’s field of work does not necessarily require the coach to possess knowledge or experience in that specific area but rather to have expertise in facilitating learning and goal attainment [54].

Informal coaching often manifests in spontaneous interactions, such as hallway conversations, brief telephone calls, or impromptu discussions over lunch. These instances serve as opportunities for supervisors to provide timely feedback and guidance to their staff as needs arise [7]. Moreover, informal coaching extends beyond the hierarchical relationship between managers and staff, as it can occur in peer-to-peer coaching within the organizational setting. Employees frequently engage in informal coaching to support, motivate, and influence each other, thereby fostering a culture of collaboration [8]. This dynamic empowers staff members to seek guidance from a diverse array of internal resources within the organization. Rather than relying solely on managerial authority, employees are encouraged to leverage the collective expertise and experiences of their colleagues to address challenges and resolve issues [4].

### Hypotheses development

#### Informal coaching and job performance.

In the social exchange assumption, it can be observed that when managers (the coaches) devote their time and effort to providing feedback and guidance for the success and growth of the coachees, the coachees may, in turn, be willing to respond by making a great effort in their performance [37]. Regarding coaching from colleagues, additional resources, an informal work environment, or a culture of mutual support provided by the organization can increase staff motivation to engage in coaching activities that support each other [44]. Accordingly, staff may also be willing to reciprocate the organization’s support by putting more effort in the job to improve their performance. Recent studies across various industries have documented the advantages of coaching in enhancing employees’ job performance. For instance, Ellinger [56] demonstrated that the coaching practices are positively correlated with employee performance in an industrial setting. Agarwal [57] stated that coaching intensity boosts sales performance. In the life insurance industry, Kim and Kuo [37] further asserted that coaching has a positive influence on the performance of subordinates compared to their counterparts. In the context of the higher education sector, we propose the following hypothesis:

*Hypothesis 1. Informal coaching from supervisor (1a) and from peers (1b) is positively related to faculty staff’s job performance.*

#### Informal coaching and POS.

POS is defined as employees’ perception of how much the company values their contributions and efforts [58]. The higher level of perceived organizational support is illustrated when employees receive coaching, as it signals the organisation’s encouragement and motivation for staff’s career growth. Similarly, additional resources, an informal work environment, or a culture of mutual support from the organization might increase staff motivation to engage in the coaching activities to support each other [44], which may lead to higher level of POS among staff. Therefore, coaching activities, in general, can be an effective way for organizations to enhance employees’ perceptions of organizational support.

To the best of our knowledge, the association between coaching and POS was found in Carrell [16] in the field of higher education in the United States. Using SET, Carrell [16] specifically endorsed the coaching activities within organizations that induce positive emotional responses among personnel and, in turn, build their perception of POS. Extending this to informal coaching, this study hypothesizes that:

*Hypothesis 2. Informal coaching from supervisor (2a) and from peers (2b) are positively related to POS.*

#### POS as a mediator.

It has been evidenced that POS is positively associated with job performance [43,59]. For instance, Eisenberger [43] argued that high levels of POS induce staff’s feelings of obligation to reciprocate by improving their job efficiency. Indeed, employees who feel recognized and supported by their organization are highly motivated to fulfill their duties to the best of their abilities [59]. Based on social exchange theory [35], increased perceived organizational support (POS) is expected to lead to greater employee commitment and a stronger sense of obligation to the organization. Therefore, by emphasizing the importance of reciprocity and mutual benefit in social relationships, social exchange theory offers the rationale that employees who receive coaching and perceive support from their organization feel more committed, which in turn increases their job performance. A few empirical studies by Grant [60] and Spence & Grant [61] suggested that subsequently leads to positive workplace outcomes. Based on the previously hypothesized relationships between informal coaching and job performance, and between informal coaching and POS, the following hypothesis is proposed:

*Hypothesis 3. POS mediates the relationship between informal coaching from supervisors (3a) and from peers (3b), and faculty staff’s job performance.*

#### Transformational leadership as a moderator.

Transformational leadership is a leadership style characterized by leaders who guide employees to understand organizational goals, and inspire them to exceed their expected requirements [27]. Additionally, transformational leaders can enhance the social exchange between the coach and coachee by providing extra resources and support beyond the coaching-based relationship [44,45]. For example, transformational leaders may offer additional training opportunities or access to developmental resources that can improve the effectiveness of coaching [45]. These resources and support can increase the employees’ motivation to engage in the coaching activities [44]. Furthermore, transformational leaders can motivate employees to continuously update their expertise [45]. As a result, a high level of transformational leadership will create a favorable working environment that inspires, supports and empowers employees, making them feel valued and encouraged to enhance their skills and abilities [62].

In the preceding discussion, we argued that informal coaching, when executed effectively, has the potential to enhance employee engagement in positive workplace behaviors, such as improved job performance and a strong perception of organizational support. However, we also acknowledged the potential role of transformational leadership in influencing these relationships. Transformational leadership involves a leader providing additional training opportunities or access to developmental resources [62]. According to social exchange theory [36], social behaviors arise from reciprocal exchanges between individuals and others in the organization. This exchange is based on the principle of reciprocity, where a transformational leader offers employees valuable resources such as care, concern, support, and a favorable work environment. As a result, employees may feel a sense of obligation, leading to positive behaviors aimed at reciprocating the actions of their leaders, colleagues and the organization. Many researchers have found transformational leadership has moderating effects on both independent and dependent variables [30–32]. In this context, we hypothesize that:

*Hypothesis 4. Transformational leadership moderates the impact of informal coaching from supervisors (4a) and from peers (4b) on faculty staff’s job performance, such that the impact at the high transformational leadership level is better than the impact at low transformational leadership level.*

Transformational leaders establish a positive work environment that fosters learning opportunities and facilitates career growth for employees [62]. Such an environment can lead to higher levels of perceived organizational support. Transformational leadership style may create a supportive working environment that, in turn, enhances the effectiveness of coaching by providing developmental resources, job recognition, and job opportunities for each coachee. This inspires them to perceive coaching as a valuable exchange, which contributes to their sense of value and importance within the organization. Drawing on these arguments, we can hypothesize that the positive relationship between informal coaching and POS will be stronger when the managers of both the coach and the coachee exhibit transformational leadership behaviors. Accordingly, the following hypothesis is proposed:

*Hypothesis 5. Transformational leadership moderates the impact of informal coaching from supervisors (5a) and from peers (5b) on POS, such that the impact at the high transformational leadership level is better than the impact at low transformational leadership level.*

**Fig 1:**
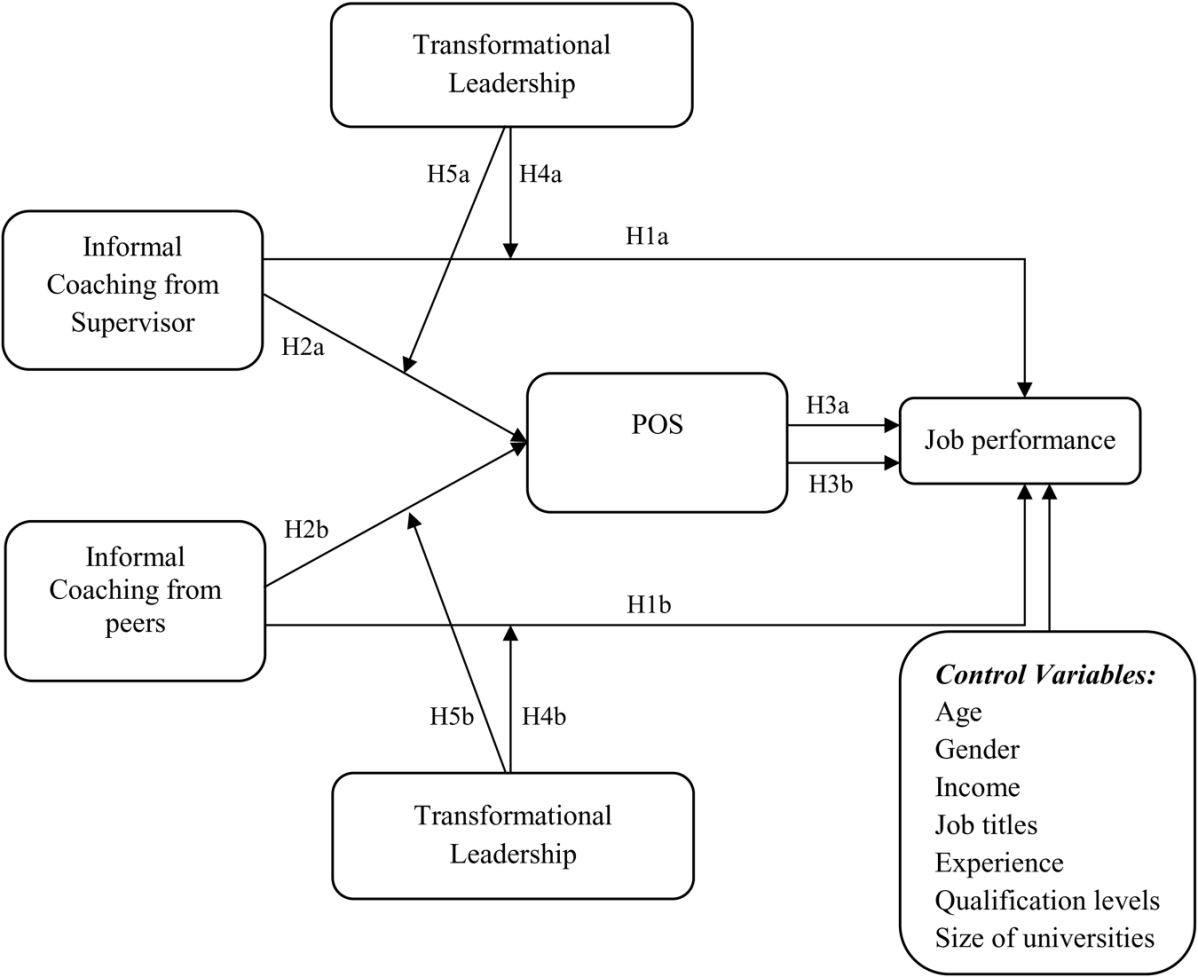
Conceptual model.

Accordingly, this research proposed the following conceptual framework as showed in fig 1:

## Materials and methods

### Research context

There were several reasons for carrying out this research in Vietnam. First, given the challenges and objectives outlined within Vietnam’s higher education sector, the implementation of informal coaching emerges as a strategic imperative. The nation’s universities, grappling with issues such as suboptimal research performance and a deficiency of Ph.D.-qualified faculty, stand to benefit significantly from this less formalized method of professional development. Informal coaching facilitates the sharing of expertise and experiences among academic staff, enhancing research skills, and fostering a culture of continuous learning and collaboration [63–66]. It enables experienced faculty to guide less seasoned colleagues in effective research methodologies, publication strategies, and teaching techniques, thereby raising both research output and teaching quality. Moreover, according to Truong [67], the rapid expansion of universities in Vietnam has heightened the demand for a robust academic workforce, making informal coaching critical in accelerating the professional growth of faculty members. This peer-driven approach not only aligns with the goals of Vietnam’s Higher Education Reform Agenda but also promotes a supportive academic community, thereby enhancing job satisfaction and reducing faculty turnover. Given the context-sensitive nature of Vietnam’s educational challenges, informal coaching offers a flexible, cost-effective solution that is particularly well-suited to the local educational landscape, ensuring that the sector’s development is both sustainable and impactful.

Second, Vietnam provides an exemplary context for exploring the effectiveness of informal coaching, given its cultural compatibility with the foundational aspects of this educational methodology. Vietnamese cultural norms, which emphasize community cohesion, respect for elders, and collaborative knowledge sharing, align seamlessly with the principles of informal coaching [65]. This cultural predisposition supports an educational environment where the exchange of knowledge is not only encouraged but also conducted within a framework that respects the hierarchical and communal values intrinsic to Vietnamese society [66]. Therefore, leveraging informal coaching in Vietnam not only taps into practical educational strategies but also integrates with cultural practices, potentially enhancing the receptivity and effectiveness of such educational interventions [67].

### Sample and data collection

Data for this study were gathered using a combination of online and on-campus surveys administered to employees working in Vietnamese higher education institutions (HEIs). A stratified random sampling method was employed to ensure that the sample accurately represented the diverse academic positions, qualifications, and tenures within the HEIs. This approach was chosen to minimize sampling bias and to provide a comprehensive understanding of the impact of informal coaching across different strata of academic staff. Stratification was based on key demographic variables such as job title, years of experience, and educational background to ensure that all relevant subgroups were adequately represented. This approach reduces the risk of sampling bias and allows for more reliable and valid conclusions regarding the impact of informal coaching within this context

The self-reported questionnaires were distributed via two channels: a Google Docs link for online responses and paper-based questionnaires for those available on-campus. The questionnaire was structured using a 7-point Likert scale, ranging from ‘strongly disagree’ to ‘strongly agree,’ which allowed for nuanced responses and helped capture the varying degrees of agreement among respondents.

To test our hypotheses, a time-lag study is used to minimize the emergence of bias by examining the responses of different academic staff at different points in time. This study gathered time-lag survey data (with a two-month interval) from 768 academic staff at various universities in Vietnam. We distributed surveys that had been translated from English to Vietnamese by a professional translator. We pre-tested the translated questionnaire with four management experts. Prior to collecting primary data, a pilot test with 90 participants was conducted to check the inaccuracies and misinterpretations of the translated questionnaire.

During the initial data collection phase, instructions were provided to explain the purpose of the study to prospective participants. Employees completed measures of informal coaching from supervisors and colleagues, as well as transformational leadership variables, at Time 1. Then, using the email addresses collected from respondents at Time 1, we sent them an online link of the Time 2 survey. They provided information about perceived organizational support and job performance variables at this time. After sending reminder notices from the Directors, we received 977 and 784 completed responses at two different times. After removing data from 15 respondents with missing information, the final sample included 768 responses. Responses were matched by the email addresses provided at both times of collection.

### Data analysis

Partial Least Squares Structural Equation Modeling (PLS-SEM) was utilized for data analysis because of its effectiveness in managing complex models with multiple constructs and its ability to reveal relationships between latent variables. The analysis was conducted using Smart-PLS software, which enabled the examination of both direct and indirect effects of informal coaching on job performance, as well as the mediating role of perceived organizational support (POS) and the moderating role of transformational leadership.

To ensure the robustness of the findings, several steps were taken during the data analysis process. First, missing data were handled using pairwise deletion, a method that retains as much data as possible by excluding cases only when the analysis involves variables with missing values. This approach was deemed appropriate due to the minimal amount of missing data. Second, multicollinearity was assessed using the Variance Inflation Factor (VIF) to ensure that the predictor variables were not highly correlated, which could otherwise distort the results. All VIF values were below the commonly accepted threshold of 5, indicating that multicollinearity was not a concern in this study.

Third, potential biases were addressed through the use of a time-lagged design and by controlling for key demographic variables that might influence the outcomes, such as age, gender, income, job title, years of experience, and the size of the university (T1). These variables were included in the analysis to account for potential confounding factors that could influence the relationship between informal coaching, POS, and job performance Additionally, bootstrapping with 5,000 resamples was performed to assess the significance of the path coefficients, further enhancing the reliability of the results. This comprehensive approach to data analysis underscores the credibility of the study’s findings and ensures that the conclusions drawn are both robust and valid

### Measurements

All constructs in the study were assessed using a Likert scale with seven points, ranging from one (strongly disagree) to seven (strongly agree).

#### Informal coaching from supervisors.

The measurement of informal coaching provided by supervisors was assessed using an 8-item Likert-type scale derived from Heslin [50]. While Heslin’s scale originally gauged coaching in a general without specification of its formal or informal type, it was tailored for this study to specifically measure informal coaching. The scale employed a response format ranging from one (strongly disagree) to seven (strongly agree). The sample items include: My direct supervisor informally “provides guidance regarding performance expectations effectively” and “helps me to analyze my performance.”

#### Informal coaching from peers.

Similarly, we also adapted an 8-item scale from Heslin [50] to assess Informal coaching from peers. The sample items include: Someone from my colleagues informally “provides guidance regarding performance expectations effectively” and “helps me to analyze my performance.”

#### Job performance.

The scale of job performance was adapted from Ozcelik & Barsade [24] to assess job performance, and consists of four items. The sample items include “I satisfactorily complete assigned duties” and “I am an effective performer.”

#### POS.

An eight-item scale by Eisenberger [68] was used to assess POS. The sample items include “My organization cares about my opinions” and “My organization really cares about my well-being”.

#### Transformational leadership.

An eight-item scale by Sun & Wang [69] was used to measure transformational leadership. The sample items include “My direct supervisor places the learning needs of staff ahead of personal and political interests” and “My direct supervisor communicates a clear vision for staff.”

#### Control variables.

According to research by Pousa & Mathieu [70] and Liu & Batt [71], the effectiveness of coaching may differ based on factors such as age, gender, income, tenure, qualification levels, and size of universities. This suggests that these factors may affect the results of the study. Additionally, other research by Liu and Batt [71], Wayne [72], and Ozcelik & Barsade [24] indicated that these variables can also influence perceptions of organizational support and job performance. Therefore, in our analyses, we accounted these factors by controlling for age, gender, income, tenure, qualification levels, and the size of universities.

#### Data bias controlling.

To mitigate the potential common method bias, this study utilized a time-lagged design. This approach, as suggested by Podsakoff [73], helps minimize the influence of common method variance on the results. Moreover, we targeted respondents with more than one year of working experience and various areas of expertise in their current institutions. This is because such respondents provide a deeper understanding of the efficiency of coaching policies adopted in the universities. This selection is consistent with the study by Pham [74]. Additionally, we also employed Harman’s single factor test to check for common method variance. Common method variance is a problem if the variance of first factor exceeds 50% of the total variance [75]. In our analysis, there are five factors with Eigenvalues higher than 1, and the overall variance for the first factor is 28,73%, less than 50%. Furthermore, this study also follows Nguyen [76] in controlling for data bias by running the measurement model of the single factor. The CFA results for measurement model are χ2 = 7603,987, df = 464, χ2/df = 16,388 > 3; GFI = 0.436 < 0.8; AGFI = 0.358 < 0.8; CFI = 0.472 < 0.9; TLI = 0.436 < 0.9; RMSEA = 0.142 > 0.08, SRMR = 0.239 > 0.08, which indicates a poor model fit. Thus, this study does not suffer from the issue of common bias.

## Results

### Demographic description

As indicated in table 1, this study sample is nearly evenly dominated between female and male (50.78% against 49.22% respectively). In terms of educational distribution, those holding a Master degree constitute a largest percentage at 41.15%. Additionally, the respondents aged between 36 and 45 are the most represented age group (31.25%), while those with less than 8 years of working experience are the largest group (32.94%). The lecturers with the income from 14 to 21 million VND mostly respond to fill out the questionnaires. We also found it easy to approach universities with a student population between 10,000 and 20,000.

**Table 1:** Demographic and descriptive information.

Criteria		Number of Respondents	Percentage
**Gender**	Female	390	50,78%
	Male	378	49,22%
**Age**	Younger than 25	97	12,63%
	From 25 to 35	198	25,78%
	From 36 to 45	240	31,25%
	From 46 to 55	129	16,79%
	Older than 55	104	13,55%
**Working experience (number of years working for the university - NYWU)**	Less than 8 years	253	32,94%
	From 8 to 15 years	214	27,86%
	From 16 to 23 years	132	17,18%
	From 24 to 31 years	87	11,32%
	More than 31 years	82	10,7%
**Qualification levels (QL)**	Undergraduate	165	21,48%
	Master degree	316	41,15%
	Doctoral degree	177	23,05%
	Others	110	14,32%
**Job titles (JT)**	Academic staff	162	21,09%
	Lecturers	255	33,20%
	Academic managers	129	16,79%
	Deans/Vice deans	116	15,10%
	Others	106	13,82%
**Income**	Less than 7 million VND	188	24,47%
	From 7 -14 million VND	186	23,66%
	From 14 -21 million VND	267	33,97%
	More than 21 million VND	127	17,9%
**Size of universities (based on number of students) (SU)**	Less than 10.000 students	198	25,78%
	From 10.000-20.000 students	379	49,35%
	More than 20.000 students	191	24,87%

### Measurement test

Nunnally and Bernstein [77] found that the outcomes (as shown in Table 2) suggest a dependable reliability, as both Cronbach’s alpha and composite reliability exceed the standard of 0.7. The current data also demonstrates adequate convergent validity, as all AVE (Average Variance Extracted) values are above 50%, meeting the requirement set by Hair [78], as detailed in Table 2.

**Table 2:** Factor analysis and reliability test.

Research Items	Factor Loading	Cronbach’s alpha	Composite Reliability	Average Variance Extracted
**ICFS (Informal coaching from supervisors)**				
ICFS1	0.770	0.907	0.910	0.605
ICFS2	0.752			
ICFS3	0.763			
ICFS4	0.786			
ICFS5	0.796			
ICFS6	0.767			
ICFS7	0.775			
ICFS8	0.810			
**ICFC (Informal coaching from peers)**				
ICFC1	0.870	0.949	0.950	0.737
ICFC2	0.850			
ICFC3	0.867			
ICFC4	0.859			
ICFC5	0.870			
ICFC6	0.849			
ICFC7	0.870			
ICFC8	0.833			
**POS (Perceived organizational support)** (*R*^2^ **= 0.230)**				
POS1	0.724	0.883	0.886	0.551
POS2	0.720			
POS3	0.764			
POS4	0.743			
POS5	0.742			
POS6	0.733			
POS7	0.750			
POS8	0.759			
**JP (Job performance) (***R*^2^ **= 0.351)**				
JP1	0.788	0.785	0.791	0.608
JP2	0.745			
JP3	0.822			
JP4	0.761			
**TL (Transformational leadership)**				
TL1	0.794	0.785	0.788	0.607
TL2	0.794			
TL3	0.778			
TL4	0.750			

To assess the discriminant validity in Table 3, it is observed that the square roots of the average variance extract (AVE) for each latent variable are higher than the correlations between any two pairs of constructs. Additionally, the AVE values for each variable are greater than the maximum shared squared variance (MSV) for that variable. Therefore, the measurement model ensures discriminant validity.

**Table 3:** Correlations among constructs.

	ICFC	ICFS	JP	POS	TL
ICFC	0.858				
ICFS	0.305^*^	0.778			
JP	0.307^**^	0.361^**^	0.780		
POS	0.294^*^	0.256^*^	0.376^**^	0.742	
TL	0.342^**^	0.296^**^	0.464^***^	0.417^**^	0.779

*Notes: Diagonal values are the square root of AVE; correlations of the constructs are below the diagnols;*

*p <  0.05,

**p <  0.01,

***p <  0.001

*(Source: Author’s calculation from research data)*

### Hypothesis testing

In this study, we utilized a time-lagged dataset and conducted PLS-SEM to analyze the conceptual model. The findings presented in Table 4 demonstrate a significant positive impact of ICFS on employee job performance (b =  0.246, p <  0.001) and POS (b =  0.144, p <  0.001). As a result, hypotheses H1A and H2A are supported, indicating that ICFS plays a crucial role in fostering positive behaviors and motivating employees to exert extra effort to enhance their work performance. Similarly, ICFC was observed to have a positive influence on both job performance (b =  0.085, p <  0.05) and POS (b =  0.159, p <  0.01). Thus, ICFC can have a range of beneficial effects, including facilitating employee learning, improving role performance, aiding individuals in achieving their work-related goals, and boosting overall morale in the workplace. These findings support hypotheses H1B and H2B.

**Table 4:** Evaluation of hypothesis testing (Direct influences).

Hypotheses	Path	Standardize Estimate	*t*-value	*p*-value	Conclusion
H1B	ICFC ⇨ JP	0.085^*^	2.198	0.028	Supported
H2B	ICFC ⇨ POS	0.159^***^	4.613	0.000	Supported
H1A	ICFS ⇨ JP	0.246^***^	6.242	0.000	Supported
H2A	ICFS ⇨ POS	0.144^***^	3.800	0.000	Supported
	POS ⇨ JP	0.158^***^	4.040	0.000	
	Age ⇨ JP	0.024	0.896	0.370	
	Gender ⇨ JP	-0.095	1.577	0.115	
	Income ⇨ JP	-0.002	0.055	0.956	
	Job titles ⇨ JP	0.051	1.749	0.081	
	Working experience ⇨ JP	0.066^*^	2.349	0.019	
	Qualification levels ⇨ JP	0.048	1.715	0.087	
	Size of universities ⇨ JP	0.024	0.905	0.366	

*p <  0.05,

**p <  0.01,

***p <  0.001.

Additionally, Table 5 shows that ICFS was found to influence JP positively and indirectly (b =  0.023, p <  0.01) via POS. Also, ICFC was found to influence JP positively and indirectly (b =  0.025, p <  0.01) via POS. Thus, employees who receive informal coaching practices from supervisors or colleagues are more likely to perceive a high level of organizational support and in turn reciprocate through increased job performance. This supported both hypotheses H3A and H3B.

**Table 5:** Evaluation of hypothesis testing (Indirect influences).

Hypotheses	Path	Standardize Estimate	*t*-value	Confidence interval	*p*-value	Conclusion
H3A	ICFS => POS => JP	0.023^**^	2.728	0.008; 0.041	0.006	Supported
H3B	ICFC => POS => JP	0.025^**^	2.973	0.012; 0.044	0.003	Supported

*p <  0.05,

**p <  0.01,

***p <  0.001.

**Fig 2:**
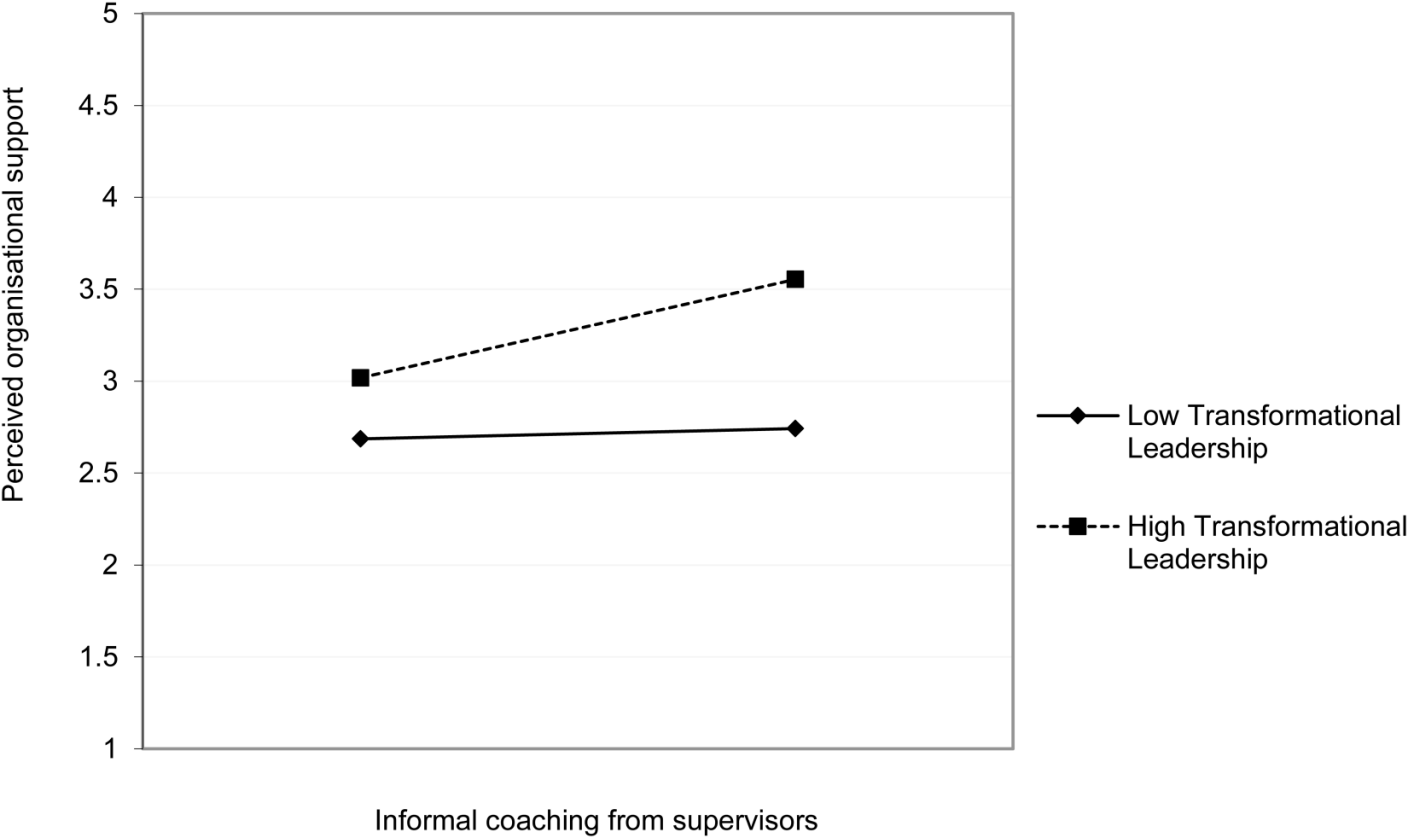
The moderating impact of transformational leadership on the connection between ICFS and POS.

**Fig 3:**
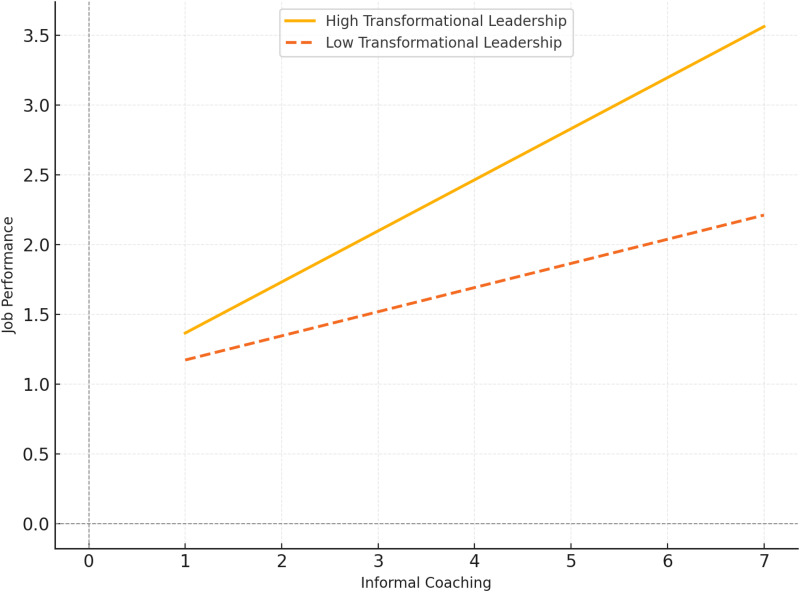
The moderating effect of transformational leadership on the connection between ICFS and JP.

Next, the results in table 6 conclude that hypotheses H5A and H4A are supported, indicating that transformational leadership moderates the connections between ICFS and POS, and ICFS and job performance (JP). Specifically, the moderating effect of transformational leadership (TL) on the path from ICFS to POS is 0.124 at p <  0.01, the path from ICFS to JP is 0.148 at p < 0.001. As illustrated in figs 2 and 3, the analysis of the moderating effect shows that when transformational leadership is at higher levels, the slope of the line is steeper and significantly surpasses the slope observed at lower levels of transformational leadership. The results from tables 7 and 8 clarified the moderating role of TL towards the influence of ICFS on POS and JP. By contrast, the analysis revealed that leadership does not exert a moderating impact on the connection between ICFC and JP, and ICFC and POS. Thus, it is concluded that H5B and H4B are not supported.

**Table 6:** Evaluation of hypothesis testing (Interactive influences).

Hypotheses	Path	Standardize Estimate	*t*-value	Confidence interval	*p*-value	Conclusion
H5A	TL x ICFS ⇨ JP	0.148^***^	3.624	0.068; 0.231	0.000	Supported
H4A	TL x ICFS ⇨ POS	0.124^**^	3.296	0.049; 0.200	0.001	Supported
H5B	TL x ICFC ⇨ JP	-0.057	1.856	-0.113; 0.006	0.071	Rejected
H4B	TL x ICFC ⇨ POS	0.033	0.950	-0.030; 0.105	0.343	Rejected

*p <  0.05,

**p <  0.01,

***p <  0.001.

**Table 7:** Conditional effect of ICFS on job performance.

Transformational leadership (TL)	Standardize Estimate	t-value	Confidence interval	p-value
Low TL	0.173^*^	2.298	-0.024; 0.291	0.022
High TL	0.366^***^	7.254	0.263; 0.462	0.000

*p <  0.05,

**p <  0.01,

***p <  0.001.

**Table 8:** Conditional effect of ICFS on POS.

Transformational leadership (TL)	Effects	t-value	Confidence interval	p-value
Low TL	0.000	0.004	-0.149; 0.166	0.997
High TL	0.249^***^	5.183	0.156; 0.342	0.000

*p <  0.05,

**p <  0.01,

***p <  0.001.

## Discussion

The result of this study has broad implications for corporate, non-profit, public sector, SMEs, and cross-cultural settings. In corporate environments, where skill development is crucial, informal coaching can be seamlessly integrated into daily operations to provide immediate feedback and development opportunities. This approach is particularly beneficial in fast-paced industries such as technology and finance, where traditional training programs may not adequately address rapidly evolving skill requirements [79]. By fostering a culture of continuous learning, informal coaching not only improves individual performance but also enhances organizational agility and competitiveness [80].

In non-profit organizations with limited resources, informal coaching presents a cost-effective alternative to formal training programs. In this context, POS plays a critical role, as employees who perceive strong organizational support are more likely to demonstrate increased commitment and lower turnover rates [81]. Transformational leadership further enhances the effectiveness of informal coaching by inspiring and empowering teams. By fostering a supportive environment, nonprofits can cultivate a dedicated workforce aligned with their mission and goals, ultimately leading to improved service delivery and greater community impact [82].

The insights from this study can also be applied to improve operational efficiency in hierarchical public sector organizations with formalized processes. Informal coaching can facilitate better communication and collaboration across departments, helping to break down silos that often impede responsiveness to community needs [83]. The study’s findings on transformational leadership provide a framework for public sector leaders to cultivate a more adaptive organizational culture in which employees feel valued and supported [84]. In such a supportive environment, employees are more likely to engage in organizational citizenship behaviors that benefit the community, thereby enhancing public service outcomes [81].

For SMEs, which may lack the resources for extensive training programs, informal coaching offers an effective method for employee development. The positive impact of informal coaching on job performance suggests that SMEs can leverage these practices to build cohesive teams and nurture talent [79]. Given the close relationships between SME leaders and their employees, personalized coaching experiences can significantly enhance motivation and performance. By prioritizing informal coaching, SMEs can promote a culture of continuous improvement and innovation, which is essential for long-term growth and sustainability [80].

When applying the findings of this study to multinational corporations operating in diverse cultural contexts, cross-cultural considerations are essential. The study’s context in Vietnam underscores the importance of understanding how cultural norms influence the effectiveness of organizational support and informal coaching [85]. Organizations that tailor their management practices to account for these cultural differences can enhance employee engagement and performance across various regions [79]. This cultural sensitivity not only improves the effectiveness of informal coaching but also strengthens employee identification and commitment, contributing to the organization’s global success [81].

### Theoretical implications

This work contributes in several crucial ways to addressing the gaps identified in the literature evaluation. First, this study highlights the distinct value of two types of informal coaching—coaching from supervisors and coaching from colleagues—in fostering a supportive work environment, each playing a unique role in shaping employee outcomes. Supervisor coaching provides authoritative guidance and aligns individual efforts with organizational goals, while peer coaching emphasizes mutual support and collaboration, enhancing team cohesion and knowledge sharing. The findings further reveal that transformational leadership moderates the relationship between supervisor coaching, job performance, and perceived organizational support (POS), but does not influence the impact of peer coaching on these outcomes. This distinction underscores the necessity of recognizing the unique dynamics underlying these coaching sources, as peer coaching operates through lateral relationships less affected by hierarchical leadership. By focusing on these distinctions and their effects on job performance, this study advances the literature on coaching [37,56,57,70,86], while addressing the limitation of generalizability in prior qualitative research through a robust quantitative approach.

Second, our study contributes to the existing literature by providing nuanced findings on how POS mediates the relationship between informal coaching from supervisors and colleagues, and employee performance in HEIs. This suggests that when employees receive informal coaching from their supervisors or colleagues, they are likely to develop a more positive perception of their organization, which in turn leads to greater job performance. This finding is consistent with previous research [20–22] that highlights the importance of POS in enhancing employee attitudes and behaviors. By uncovering one under-studied mediator (e.g., POS) in this relationship, this study extends SET [35] by incorporating POS as a new factor to elucidate the behavioral connection between the coaches and coachees in the context of HEIs in a developing country like Vietnam.

Third, by developing a model specifically designed to investigate the interactive impact of transformational leadership, our study enhances our understanding of how informal coaching from supervisors affects employee outcomes and perceptions. Our findings emphasize the significance of transformational leadership, highlighting that when employees work in an organization with a higher number of transformational leaders, informal coaching from supervisors becomes more effective in influencing their behaviors and perceptions within the workplace. Compared to recent publications on the topic of transformational leadership and employee behaviors [30], there is a scarcity of empirical research investigating the role of transformational leadership in relation to informal coaching. Consequently, gaining a deeper comprehension of how transformational leaders shape the outcomes of informal coaching from supervisors can fill existing research gaps and contribute to the current theoretical knowledge.

Regrettably, our research findings indicate that transformational leadership does not exert a moderating effect on the association between informal coaching from peers and employee performance, nor between informal coaching from peers and the perception of organizational support. Transformational leadership involves leaders guiding their employees to understand the organizational goals and inspiring them to exceed their expected requirements [27] by creating a supportive working environment that, in turn, enhances the effectiveness of coaching [25]. Thus, transformational leadership is an essential factor in improving the effectiveness of coaching behaviors and leading to higher levels of perceived organizational support, as staff perceive coaching as a valuable exchange from leaders, contributing to their sense of value and importance within the organization.

At the same time, informal coaching from peers may rely mainly on the willingness of both coach and coachee to assist each other in resolving problems as they arise. Hence, while establishing a supportive work environment is crucial for facilitating the effectiveness of informal coaching practices and achieving high levels of performance and perceived organizational support among employees, our findings suggest that employees who operate under transformational leaders and within organizations that prioritize informal coaching are more likely to engage in behaviors beneficial for informal coaching from supervisors rather than from colleagues. Therefore, we did not find evidence to support the notion that the association between informal coaching from colleagues and staff performance, as well as the relationship between informal coaching from peers and perceived organizational support, is significantly influenced by the presence of transformational leaders.

Finally, this study contributes to the current literature by examining the influence of informal coaching on staff performance in a new research area, namely higher education sector, which is a paucity of research on the application of informal coaching into HEIs in one developing country. This study assumes that its findings can be broadly applied in other developing countries.

### Practical implications

The following key benefits highlight the contributions for HEIs that plan to adopt effective informal coaching practices within their institutions.

To successfully integrate informal coaching, HEIs should first create an environment that encourages continuous learning and open communication. This can be achieved by promoting regular, informal interactions between supervisors, colleagues, and faculty members. Institutions might consider establishing mentorship programs, peer-coaching groups, or regular informal check-ins focused on both personal and professional development. These initiatives can help normalize the practice of informal coaching, making it a natural part of the organizational culture. Research has shown that mentorship serves as a beneficial tool for faculty, particularly for those from underrepresented backgrounds, by providing essential support and guidance in navigating academic challenges [87].

Moreover, to cultivate transformational leadership, HEIs should invest in targeted leadership development programs that focus on the key aspects of transformational leadership: inspirational motivation, individualized consideration, intellectual stimulation, and idealized influence. Workshops and training sessions should be designed to equip leaders with the skills necessary to inspire and empower their teams. For instance, leadership training might include simulations and role-playing exercises that prepare leaders to handle real-world challenges while motivating and intellectually engaging their staff. Such training programs can significantly enhance the capacity of academic leaders to foster an environment conducive to informal coaching and continuous improvement [88].

In addition, HEIs should incorporate transformational leadership behaviors into performance evaluation criteria for academic and administrative leaders. By aligning evaluations with leadership behaviors that prioritize the growth and development of employees, institutions can incentivize leaders to adopt and practice transformational leadership more consistently. This alignment not only reinforces the importance of effective leadership but also ensures that leaders are held accountable for fostering a supportive environment that encourages informal coaching and professional development [89]. Finally, recognizing and rewarding effective informal coaching and transformational leadership is crucial. HEIs can establish recognition programs that highlight and reward leaders and staff who excel in these areas, thereby reinforcing the importance of these practices and encouraging their widespread adoption. By institutionalizing these practices, HEIs can foster a culture that values continuous learning and effective leadership, ultimately leading to enhanced faculty performance and organizational success. Such recognition can motivate individuals to engage more deeply in mentoring relationships and informal coaching, further embedding these practices into the institutional culture [90].

## Limitations and suggestions for future research

Firstly, while the current study examines the direct and moderating effects of transformational leadership on the relationship between informal coaching and job performance, the potential for moderated mediation was not initially explored. Moderated mediation occurs when the mediating effect of a variable (e.g., POS) on the relationship between an independent variable (e.g., informal coaching) and a dependent variable (e.g., job performance) is contingent upon the level of a moderator (e.g., transformational leadership). Therefore, we suggest in future research exploring such moderated mediation effects, particularly how transformational leadership might influence the strength of POS as a mediator in the informal coaching-job performance relationship. This would provide a more comprehensive understanding of the dynamics at play.

Secondly, a limitation of this study is its focus on short-term outcomes, leaving the long-term impact of informal coaching and the potential influences of various mediators and moderators unexplored. Future research should build on this study by examining the long-term effects of informal coaching on outcomes such as job performance, employee retention, career progression, and overall organizational effectiveness. Longitudinal studies, which track these variables over time, would be particularly valuable in establishing causal relationships and understanding whether the benefits of informal coaching are sustained, amplified, or diminished as employees advance in their careers. Additionally, future research should explore the role of mediators and moderators, such as organizational culture factors—like openness, supportiveness, and innovation—and individual personality traits, including openness to experience, conscientiousness, and emotional stability. Understanding how these factors influence the effectiveness of informal coaching would provide insights into the optimal conditions for its success and help tailor coaching practices to various organizational settings.

Thirdly, this study focuses on a HEIs context, which may not capture the full range of factors influencing informal coaching’s effectiveness. Future research should explore the comparative effectiveness of informal coaching across different organizational contexts, such as corporate, non-profit, and public sector settings, as well as across diverse cultures and regions. Such studies would provide valuable insights into situational factors that influence coaching success and help develop context-sensitive practices. Additionally, investigating the interaction between informal and formal coaching programs could inform better employee development strategies by determining whether these approaches are complementary or more effective when combined. Finally, expanding the scope of outcomes studied—including well-being, job satisfaction, creativity, and organizational citizenship behavior—would offer a more comprehensive understanding of informal coaching’s impact on both individuals and organizations, advancing both theory and practice.

Lastly, this study is conducted in Vietnam, which may limit the generalizability of the findings to other cultural contexts. Vietnam’s unique cultural characteristics—such as high power distance, collectivism, and moderate uncertainty avoidance—likely influenced how informal coaching, POS, and transformational leadership were perceived and practiced [49]. These cultural factors may affect the effectiveness of these constructs differently compared to other regions, particularly in developed economies or cultures with contrasting values, such as lower power distance or greater individualism [91]. Consequently, the findings from this study may not fully translate to settings where organizational structures, social norms, and leadership expectations differ significantly. Future research should therefore investigate cross-cultural differences in the effectiveness of informal coaching, POS, and transformational leadership. Conducting comparative studies across diverse cultural contexts would offer valuable insights into how these management practices operate in various environments, potentially identifying cultural moderators that influence their success [92].

## Supporting information

S1 Appendix Descriptive analysis for items. (DOCX)
