# Supplementary material for: Employee informal coaching and job performance in higher education: The role of perceived organizational support and transformational leadership
Source: PLoS One. 2025 Apr 1;20(4):e0320577. doi: 10.1371/journal.pone.0320577 (PMC11960931; doi:10.1371/journal.pone.0320577)
Supplement: S1 Appendix — (DOCX) [file pone.0320577.s001.docx]

**Appendix**

# **Descriptive Analysis for Items.**

| **Research Items** | **Mean** | **Std. Dev.** | **Sources** |
| --- | --- | --- | --- |
| **ICFS (Informal coaching from supervisors)**  **My direct supervisor informally….** |  |  | Heslin (2006) |
| 1. “provides guidance regarding performance expectations effectively.” | 5.581 | 1.139 |  |
| 2. “helps me to analyze my performance.” | 5.521 | 1.108 |  |
| 3. “acts as a sounding board for me to develop my ideas.” | 5.544 | 1.085 |  |
| 4. “facilitates creative thinking to help solve problems.” | 5.585 | 1.123 |  |
| 5. “encourages me to explore and try out new alternatives.” | 5.626 | 1.047 |  |
| 6. “expresses confidence that I can develop and improve.” | 5.690 | 1.068 |  |
| 7. “encourages me to continuously develop and improve.” | 5.685 | 1.086 |  |
| 8. “supports me in taking on new challenges.” | 5.607 | 1.118 |  |
| **ICFC (Informal coaching from colleagues)**  **Someone from my colleagues informally…** |  |  | Heslin (2006) |
| 1. “provides guidance regarding performance expectations effectively.” | 5.638 | 1.094 |  |
| 2. “helps me to analyze my performance.” | 5.579 | 1.069 |  |
| 3. “acts as a sounding board for me to develop my ideas.” | 5.638 | 1.106 |  |
| 4. “encourages creative thinking to help solve problems.” | 5.669 | 1.059 |  |
| 5. “encourages me to explore and try out new alternatives.” | 5.629 | 1.058 |  |
| 6. “expresses confidence that I can develop and improve.” | 5.729 | 1.020 |  |
| 7. “encourages me to continuously develop and improve.” | 5.634 | 1.078 |  |
| 8. “supports me in taking on new challenges.” | 5.720 | 0.987 |  |
| **POS (Perceived organizational support)** |  |  | Eisenberger et al. (1997) |
| 1. “My organization cares about my opinions.” | 5.354 | 1.269 |  |
| 2. “My organization really cares about my well-being.” | 5.240 | 1.314 |  |
| 3. “My organization strongly considers my goals and values.” | 5.236 | 1.288 |  |
| 4. “Help is available from my organization when I have a problem.” | 5.345 | 1.268 |  |
| 5. “My organization would forgive an honest mistake on my part.” | 5.280 | 1.325 |  |
| 6. “If given the opportunity, my organization would not take advantage of me.” | 5.207 | 1.303 |  |
| 7. “My organization shows much concern for me.” | 5.233 | 1.300 |  |
| 8. “My organization is willing to help me if I need a special favor.” | 5.342 | 1.278 |  |
| **JP (Job performance)** |  |  | Ozcelik & Barsade (2018) |
| 1. “I satisfactorily complete assigned duties.” | 5.600 | 1.162 |  |
| 2. “I am an effective performer.” | 5.721 | 1.074 |  |
| 3. “I am a good individual contributor.” | 5.634 | 1.189 |  |
| 4. “I respond to the needs of others in my workplace.” | 5.672 | 1.118 |  |
| **TL (Transformational leadership)** |  |  | Sun & Wang (2016) |
| 1. “My direct supervisor places the learning needs of staff ahead of personal and political interests.” | 5.378 | 1.229 |  |
| 2. “My direct supervisor communicates a clear vision for staff.” | 5.499 | 1.189 |  |
| 3. “My direct supervisor encourages open communication on important organization’s issues.” | 5.573 | 1.175 |  |
| 4. “It is okay to discuss feelings, worries, and frustrations with my direct supervisor.” | 5.499 | 1.177 |  |
